# Dupilumab Effects on Innate Lymphoid Cell and Helper T Cell Populations in Patients with Atopic Dermatitis

**DOI:** 10.1016/j.xjidi.2021.100003

**Published:** 2021-02-20

**Authors:** Yasutomo Imai, Minori Kusakabe, Makoto Nagai, Koubun Yasuda, Kiyofumi Yamanishi

**Affiliations:** 1 Department of Dermatology, Hyogo College of Medicine, Nishinomiya, Japan; 2 Department of Immunology, Hyogo College of Medicine, Nishinomiya, Japan

**Keywords:** AD, atopic dermatitis, ILC, innate lymphoid cell, scRNA-seq, single-cell RNA sequencing, Th, T helper type

## Abstract

Group 2 innate lymphoid cells (ILCs) are thought to contribute to the pathogenesis of atopic dermatitis (AD). IL-4 stimulates T helper type 2 (Th2) cells and ILC2s to proliferate and produce cytokines. Dupilumab, an antibody against the IL-4 receptor, is used in AD therapy. We speculated that its efficacy might involve blocking the activation of Th2 cells and ILC2s via IL-4. Here, we examined circulating Th2 cells and ILC2s in 27 Japanese patients with AD before and after the administration of dupilumab. Between 0 and 4 months after dupilumab administration, the percentages of Th2 cells and ILC2s were decreased. Notably, ILC2/3 ratio was decreased after dupilumab treatment. Interestingly, ILC2/3 ratio before dupilumab treatment were significantly higher in high responders than in low responders to dupilumab. To resolve the molecular signatures of the Th2 and ILC2s in AD, we sorted CD4^+^ T cells and ILCs from peripheral blood and analyzed their transcriptomes using the BD Rhapsody Single-cell RNA sequencing system. Between 0 and 4 months after dupilumab administration, the Th2 and ILC2 cluster gene signatures were downregulated. Thus, dupilumab might improve dermatitis by suppressing the Th2 cell and ILC2 populations and altering the Th2 and ILC2 repertoire in patients with AD.

## Graphical abstract

**Figure:**
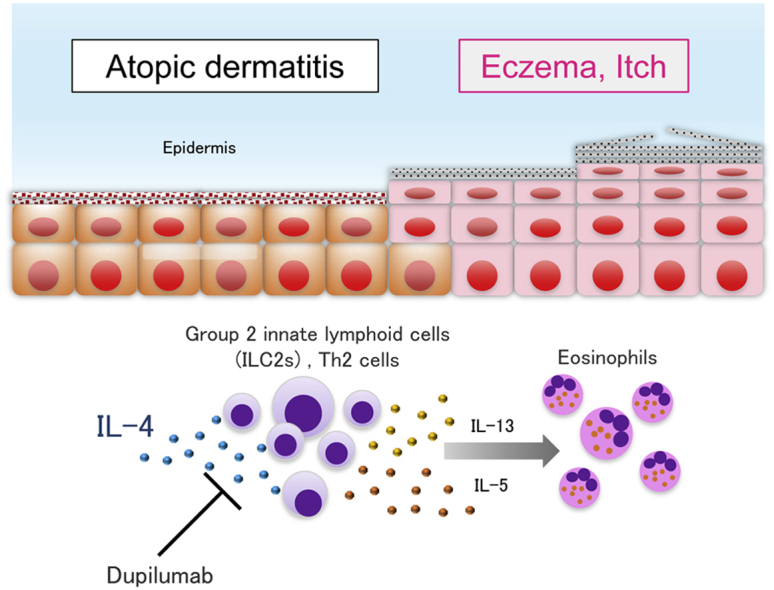

## Introduction

Group 2 innate lymphoid cells (ILCs) are thought to contribute to the pathogenesis of atopic dermatitis (AD) (Imai, 2019). ILC1s (NK cells), ILC2s, and ILC3s mirror the corresponding T helper type (Th) 1, Th2, and Th17 cells, respectively. Previously, we and others reported that IL-4 stimulates Th2 cells and ILC2s to proliferate and produce type 2 cytokines (Imai et al., 2019; Motomura et al., 2014). Dupilumab, an antibody against IL-4 receptors, is widely used in AD therapy (Beck et al., 2014; Guttman-Yassky et al., 2019), although we speculated that its efficacy in AD treatment might involve blocking the proliferation and activation of Th2 cells and ILC2s via IL-4. A recent report demonstrated a gradual decrease in the percentage of Th2 cells after dupilumab treatment (Trichot et al., 2021). However, the effects of dupilumab on ILC2s are not fully understood.

## Result

**Figure 1:**
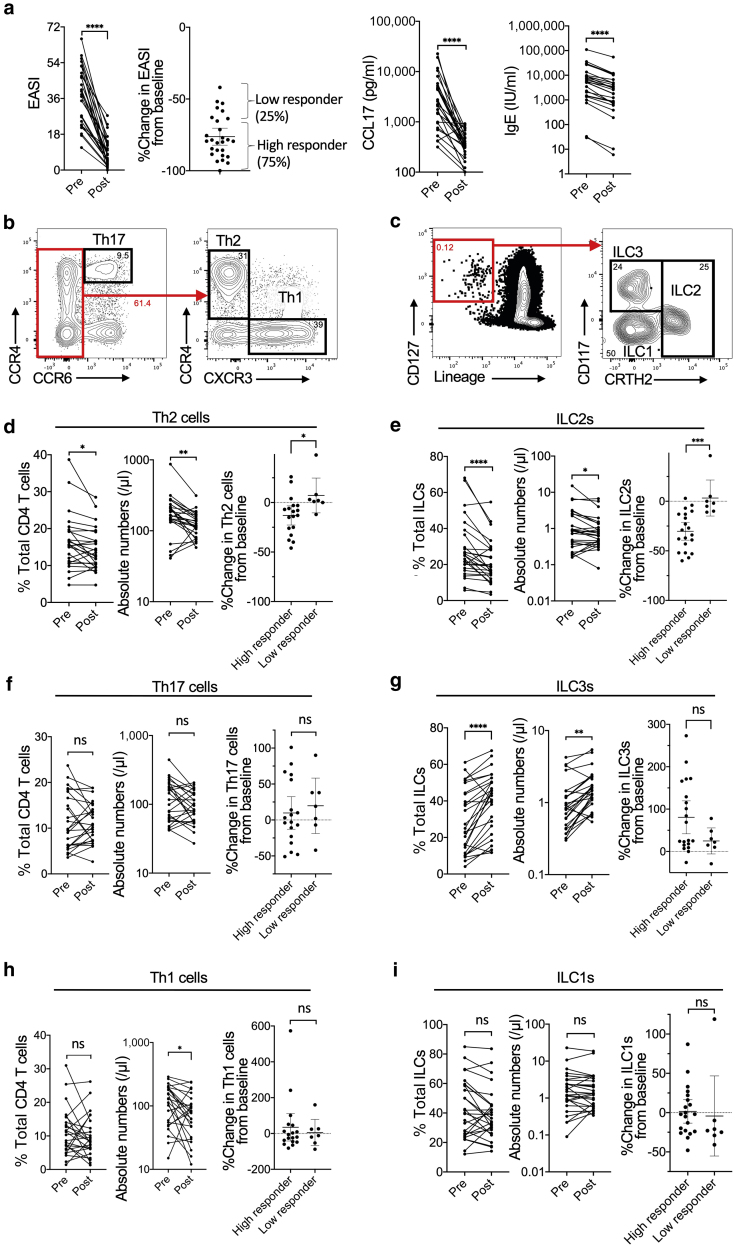
**Changes in clinical data, Th cell populations, and ILC populations before and 16 weeks after initial dupilumab treatment.** (**a**) EASI score of each patient (Pre and Post) and % change from baseline of EASI at week 16. Definition of high responder (75% of total patients) and low responder (25%) of dupilumab is shown. (**b**) Gating strategy to identify Th2 and Th17 cells. (**c**) Gating strategy to identify ILC2s and ILC3s. (**d**) Percentages of Th2 cells among CD4^+^ T cells, absolute numbers, and % change in Th2 cells from baseline, before (Pre) and 16 weeks after (Post) the dupilumab treatment. (**e**) Percentages of ILC2s among total ILCs, absolute numbers of ILC2s, and % change in ILC2s from baseline. (**f**) Percentages of Th17 cells among CD4^+^ T cells, absolute numbers of Th17 cells, and % change in Th17 cells from baseline. (**g**) Percentages of ILC3s among total ILCs, absolute numbers of ILC3s, and % change in ILC3s from baseline. Wilcoxon matched-pairs signed rank test or Mann-Whitney test was used to assess statistical significance. ∗∗∗∗*P* < 0.0001; ∗∗∗*P* < 0.001; ∗∗*P* < 0.01; ∗*P* < 0.05. Each dot represents a value for each patient. Bold lines represent estimated mean and thin lines indicate the 95% confidence interval. EASI, Eczema Area and Severity Index; ILC, innate lymphoid cell; ns, not significant; Th, T helper type.

In this study, we examined the proportion and number of circulating Th2 cells and ILC2s in 27 Japanese patients with AD before and after dupilumab administration (details of patient information are provided in Table 1). Treatment began with a 600-mg loading dose of dupilumab, followed by 300 mg dupilumab every other week, combined with topical corticosteroids and/or tacrolimus. Eczema Area and Severity Index was measured to assess disease activity, and blood was collected at the baseline visit (before therapy) and week 16, with a window of assessment of approximately 7 days. Dupilumab treatment significantly improved dermatitis and decreased total serum IgE and serum CCL17 (also known as TARC) levels in patients with AD, although some patients were low responders (Figure 1a). We used flow cytometry to assess the populations of Th2 cells (CD4^+^, CCR6^−^, CXCR3^−^, and CCR4^+^) and ILC2s (Lin^−^, CD127^+^, and CRTH2^+^) (Figure 1b and c). Between 0 and 4 months after dupilumab administration, the percentage of Th2 cells (among total CD4^+^ T cells) and ILC2s (among all ILCs) were decreased from 16.4 ± 7.6% to 14.5 ± 5.8% (mean ± SD) and 28.1 ± 16.1% to 21.2 ± 12.1%, respectively (Figure 1d and e). The absolute numbers of Th2 cells and ILC2s were significantly decreased. Both Th2 cells and ILC2s were more depleted in high responders than in low responders to dupilumab, suggesting that these cells are important in IL-4 receptor signaling pathways in AD pathogenesis. The percentage and absolute number of Th17 cells tended to increase, although this was not statistically significant (Figure 1f). The percentage and absolute number of ILC3s increased significantly after dupilumab treatment (Figure 1g). The absolute numbers of Th1 cells slightly tended to decrease (Figure 1h), and the percentage and absolute number of ILC1s were unchanged after dupilumab treatment (Figure 1i).

**Table 1:** Th2/17 and ILC2/3 Cell Percentages and Clinical Information about Each Patient

							% Total CD4 T Cells				Absolute Numbers (/μl)			% Change from Baseline			% Total ILCs				Absolute Numbers (/μl)			% Change from Baseline				
Timepoint (Weeks)	AD Patient ID	Gender	Age	IGA	EASI	% Change in EASI from Baseline	Th2	Th1	Th17	Th2/17 ratio	Th2	Th1	Th17	Th2	Th1	Th17	ILC2	ILC1	ILC3	ILC2/3 ratio	ILC2	ILC1	ILC3	ILC2	ILC1	ILC3	TARC (pg/ml)	Total IgE (IU/ml)
0	924	M	48	3	39.6	−89.4	12.0	12.7	9.8	1.23	213	226	173	20.8	−20.5	63.1	29.5	50.0	20.5	1.44	0.88	1.50	0.61	−42.9	−27.3	128.6	11,680	27,100
16				1	4.2		14.5	10.1	15.9	0.91	188	131	206				16.9	36.4	46.8	0.36	0.62	1.33	1.71				779	11,100
0	929	M	38	4	46.6	−100.0	15.7	11.2	11.5	1.37	270	193	198	−24.8	−44.8	−7.0	25.0	50.0	25.0	1.00	1.18	2.36	1.18	−37.3	−31.7	100.8	950	3460
16				0	0		11.8	6.2	10.7	1.10	161	84	146				15.7	34.1	50.2	0.31	0.73	1.58	2.33				161	697
0	934	M	28	4	52.4	−93.5	19.7	25.4	6.2	3.16	207	267	65	−46.2	3.1	7.1	68.1	27.8	4.1	16.46	15.07	6.14	0.92	−37.9	52.3	272.8	10,510	2,060
16				1	3.4		10.6	26.2	6.7	1.59	75	186	47				42.3	42.3	15.4	2.74	4.00	4.00	1.46				256	1,230
0	902	M	23	4	46.5	−92.7	10.7	7.3	3.6	2.98	166	112	56	26.2	−82.3	67.4	52.9	39.9	7.2	7.36	6.22	4.68	0.84	3.4	−19.5	83.7	5,777	29,300
16				2	3.4		13.5	1.3	6.0	2.25	126	12	56				54.7	32.1	13.2	4.14	6.68	3.92	1.61				329	10,300
0	909	F	60	4	52.2	−94.6	16.9	7.1	5.7	2.95	132	55	45	−3.0	−6.2	78.3	21.7	62.0	16.3	1.33	0.42	1.20	0.32	−53.4	−26.2	171.0	19,140	1,480
16				1	2.8		16.4	6.6	10.2	1.61	118	48	73				10.1	45.7	44.2	0.23	0.30	1.35	1.30				489	889
0	923	M	49	4	42.9	−71.3	15.0	21.1	19.5	0.77	140	197	182	−14.7	−64.5	−22.6	21.6	55.4	23.0	0.94	0.92	2.37	0.98	−13.3	−5.2	25.1	1,459	1,000
16				2	12.3		12.8	7.5	15.1	0.85	81	47	96				18.8	52.5	28.8	0.65	0.75	2.10	1.15				587	383
0	930	M	52	4	22.7	−78.4	24.8	15.7	14.2	1.75	315	199	180	−9.3	−47.8	−48.2	5.7	85.0	9.3	0.62	0.72	10.74	1.17	−17.2	−1.7	26.2	4,760	35,800
16				1	4.9		22.5	8.2	7.4	3.06	169	62	55				4.7	83.6	11.7	0.40	0.93	16.47	2.31				467	12,000
0	938	M	42	3	26.7	−84.6	13.1	11.6	14.9	0.88	145	128	165	−38.2	−26.4	−51.0	14.8	69.3	15.9	0.93	0.85	4.00	0.92	−4.4	6.9	−26.1	696	6,380
16				2	4.1		8.1	8.5	7.3	1.11	93	99	84				14.1	74.1	11.8	1.20	0.65	3.42	0.54				343	3,340
0	895	M	23	3	17.4	−88.5	21.9	10.3	21.1	1.04	218	102	210	−6.4	8.7	−10.9	18.0	59.5	22.5	0.80	0.93	3.08	1.17	−7.5	−23.9	69.1	755	1,350
16				1	2		20.5	11.2	18.8	1.09	58	32	54				16.7	45.2	38.1	0.44	1.07	2.90	2.44				315	606
0	900	M	29	3	27.3	−80.6	No data										66.7	23.4	9.9	6.73	2.48	0.87	0.37	−34.2	27.3	165.6	526	13,500
16				0	5.3												43.9	29.8	26.3	1.67	1.95	1.33	1.17				195	4,840
0	918	M	38	3	37	−79.5	19.2	8.0	18.7	1.03	271	113	263	−13.0	−24.4	−3.7	49.0	39.5	11.5	4.26	2.99	2.41	0.70	−38.8	−1.9	171.6	4,459	7,760
16				2	7.6		16.7	6.1	18.0	0.93	151	55	162				30.0	38.8	31.3	0.96	1.53	1.98	1.60				410	4,120
0	926	M	57	4	54.6	−85.7	20.5	13.2	18.0	1.14	279	179	245	−6.8	−37.6	−31.7	7.0	41.9	51.1	0.14	0.20	1.19	1.45	−51.9	0.4	6.8	419	5,180
16				2	7.8		19.1	8.2	12.3	1.55	179	77	115				3.4	42.1	54.5	0.06	0.08	0.99	1.28				865	3,520
0	932	M	42	4	66	−79.4	No data										12.4	77.5	10.1	1.23	0.37	2.31	0.30	−12.7	−12.9	115.0	8,066	110,000
16				2	13.6												10.8	67.5	21.7	0.50	0.36	2.25	0.72				681	54,600
0	939	M	49	4	57.6	−62.8	32.5	5.9	16.6	1.96	870	157	444	−12.3	24.1	−7.2	20.1	69.6	10.3	1.96	6.61	22.84	3.37	−5.3	−10.0	78.0	4,380	9,200
16				3	21.4		28.5	7.3	15.4	1.85	313	80	169				19.1	62.7	18.3	1.04	5.66	18.61	5.42				341	2,920
0	942	M	25	4	40.4	−80.0	16.8	8.6	6.3	2.68	165	84	62	−26.2	12.4	14.0	36.5	24.0	39.6	0.92	2.59	1.70	2.82	−56.8	87.2	−0.5	2,154	29.3
16				1	8.1		12.4	9.6	7.2	1.73	136	106	79				15.8	44.8	39.4	0.40	1.39	3.94	3.46				308	10.3
0	947	M	42	3	11.3	−90.3	11.2	15.6	12.3	0.91	207	288	227	−8.0	46.2	9.8	43.4	12.1	44.4	0.98	3.18	0.89	3.25	−23.3	16.3	18.3	414	1,430
16				1	1.1		10.3	22.8	13.5	0.76	106	234	138				33.3	14.1	52.6	0.63	1.35	0.57	2.12				122	830
0	954	M	30	3	38	−63.7	6.5	4.2	5.4	1.22	51	33	42	47.8	−19.7	37.6	20.0	28.0	52.0	0.38	0.31	0.43	0.80	0.0	−28.6	15.4	4,634	9,020
16				3	13.8		9.7	3.4	7.4	1.31	77	27	59				20.0	20.0	60.0	0.33	0.44	0.44	1.32				373	4,890
0	953	F	52	4	52.4	−56.1	15.4	31.0	18.4	0.84	65	131	78	3.2	−53.2	2.2	28.6	14.3	57.1	0.50	0.18	0.09	0.37	−1.6	118.8	−28.9	2,078	5,070
16				2	23		15.9	14.5	18.8	0.85	70	63	82				28.1	31.3	40.6	0.69	0.47	0.52	0.67				370	1,770
0	994	M	44	4	48.8	−65.4	16.4	1.4	4.2	3.87	185	15	48	−14.0	572.6	101.2	25.0	25.0	50.0	0.50	0.22	0.22	0.44	−51.5	33.3	9.1	988	2,310
16				2	16.9		14.1	9.1	8.5	1.65	168	108	102				12.1	33.3	54.5	0.22	0.26	0.71	1.16				920	774
0	965	M	41	4	56.1	−77.0	38.7	7.1	23.7	1.63	236	43	145	−32.8	−59.7	−45.1	26.8	58.5	14.6	1.83	0.91	1.98	0.49	−9.6	−48.2	210.6	22,660	29,500
16				2	12.9		26.0	2.9	13.0	2.00	144	16	72				24.2	30.3	45.5	0.53	0.51	0.64	0.96				224	11,600
0	1021	M	38	3	47.2	−41.9	15.3	5.0	9.8	1.56	154	50	99	8.5	25.9	38.5	16.7	22.2	61.1	0.27	0.16	0.22	0.60	−10.0	−21.2	10.5	5,196	7,230
16				3	27.4		16.6	6.3	13.6	1.22	214	81	176				15.0	17.5	67.5	0.22	0.33	0.39	1.49				566	3,000
0	980	M	43	4	34.8	−58.3	4.7	16.3	4.6	1.03	45	153	43	−0.2	−87.9	−41.8	13.6	59.1	27.3	0.50	0.19	0.81	0.37	4.8	−23.4	48.4	1,756	5,160
16				2	14.5		4.7	2.0	2.7	1.77	67	20	27				14.3	45.2	40.5	0.35	0.38	1.22	1.09				100	1,820
0	1002	M	43	3	18.2	−85.7	9.8	2.3	6.4	1.54	41	26	70	−3.2	100.9	−2.5	39.1	26.1	34.8	1.13	0.48	0.32	0.43	−27.0	9.5	23.2	2,217	9,750
16				2	2.6		9.5	4.7	6.2	1.53	115	30	39				28.6	28.6	42.9	0.67	0.46	0.46	0.69				738	6,680
0	1049	M	34	3	21.3	−53.1	19.3	6.2	7.7	2.51	148	48	59	1.0	160.0	19.1	20.0	42.5	37.5	0.53	0.44	0.95	0.83	45.8	−41.2	22.2	2,756	5,730
16				2	10		19.5	16.2	9.2	2.13	112	93	53				29.2	25.0	45.8	0.64	0.40	0.34	0.63				257	2,590
0	1062	F	29	3	24.5	−75.5	8.3	11.6	9.0	0.92	147	205	159	4.3	50.9	56.3	23.1	33.1	43.8	0.53	2.24	3.20	4.25	−60.2	22.3	15.0	316	32.9
16				2	6		8.7	17.5	14.1	0.62	119	239	193				9.2	40.4	50.4	0.18	0.91	3.98	4.96				101	6
0	945	M	46	3	22.4	−51.8	10.5	14.0	5.8	1.83	147	195	80	1.9	−12.1	89.6	26.7	35.0	38.3	0.70	0.86	1.13	1.23	−10.7	−25.2	30.4	1,513	8,710
16				1	10.8		10.7	12.3	10.9	0.98	140	161	143				23.8	26.2	50.0	0.48	0.83	0.91	1.74				346	4,930
0	1058	M	28	3	22.4	−78.6	15.7	4.8	9.5	1.66	194	59	117	−39.9	241.0	2.6	26.0	22.0	52.0	0.50	0.73	0.62	1.46	−29.0	−23.1	24.3	2,561	757
16				1	4.8		9.4	16.3	9.7	0.97	112	194	116				18.5	16.9	64.6	0.29	0.64	0.59	2.26				318	191

Abbreviations: AD, atopic dermatitis; EASI, Eczema Area and Severity Index; F, Female; ID, identification; IGA, Investigator Global Assessment; ILC, innate lymphoid cell; M, male; Th, T helper type.

**Figure 2:**
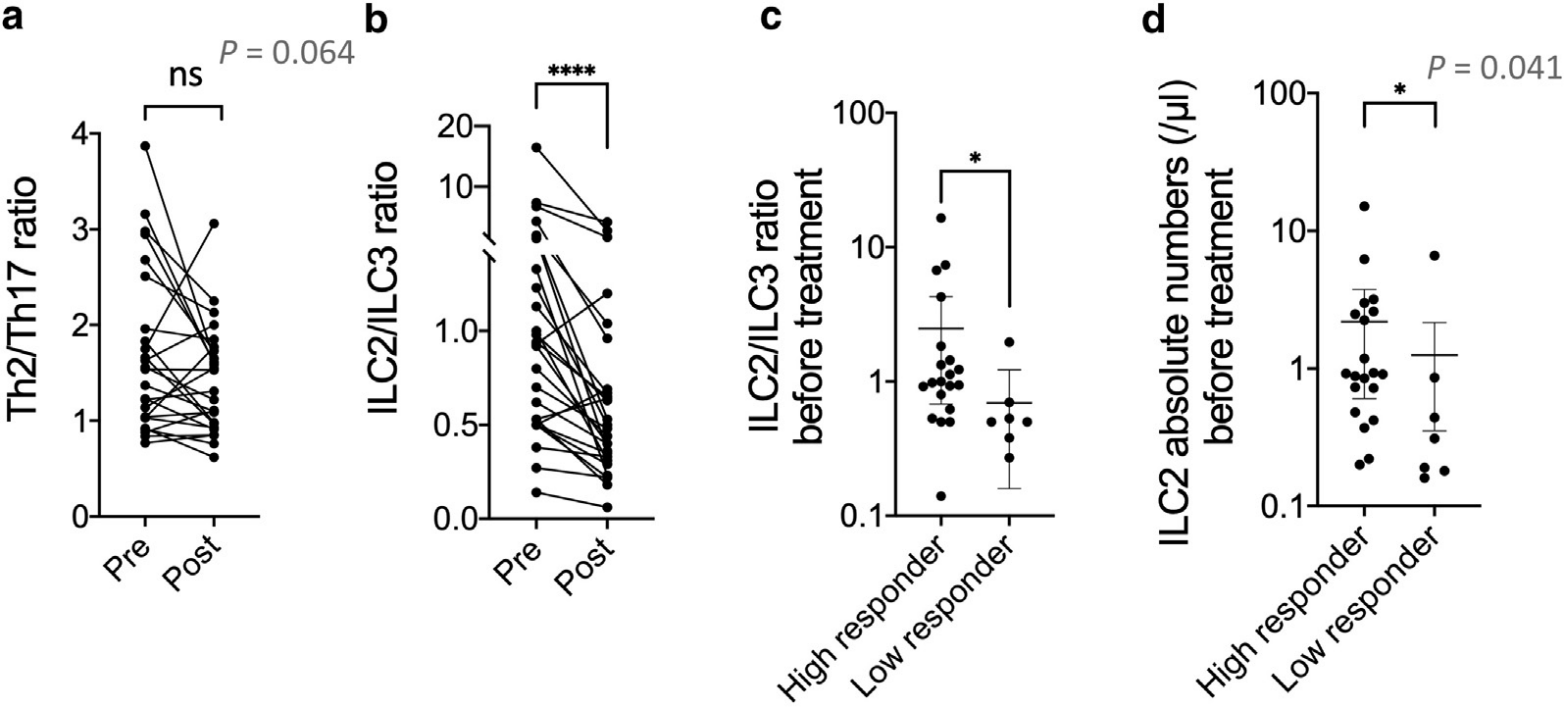
**Involvement of ILC2/ILC3 balance in treatment with dupilumab.** (**a**) Th2/Th17 ratio before (Pre) and 16 weeks after (Post) dupilumab treatment. (**b**) ILC2/ILC3 ratio before (Pre) and 16 weeks after (Post) dupilumab treatment. (**c**) ILC2/ILC3 ratio before administration of dupilumab. (**d**) Absolute numbers of ILC2s before administration of dupilumab. Wilcoxon matched-pairs signed rank test or Mann-Whitney test was used to assess statistical significance. ∗∗∗∗*P* < 0.0001; ∗*P* < 0.05. Each dot represents a value for each patient. Bold lines represent estimated mean and thin lines indicate the 95% confidence interval. ILC, innate lymphoid cell; ns, not significant; Th, T helper type.

Next, we examined Th2/17 and ILC2/3 balance. Th2/Th17 ratio tended to decrease, although it was not statistically significant (Figure 2a). ILC2/3 ratio was very significantly decreased after dupilumab treatment (Figure 2b). ILC2/3 ratio and the absolute numbers of ILC2s before dupilumab treatment were significantly higher in high responders than in low responders to dupilumab (Figure 2c and d), suggesting that dupilumab might be more effective in patients whose immune balance is originally skewed toward ILC2.

**Figure 3:**
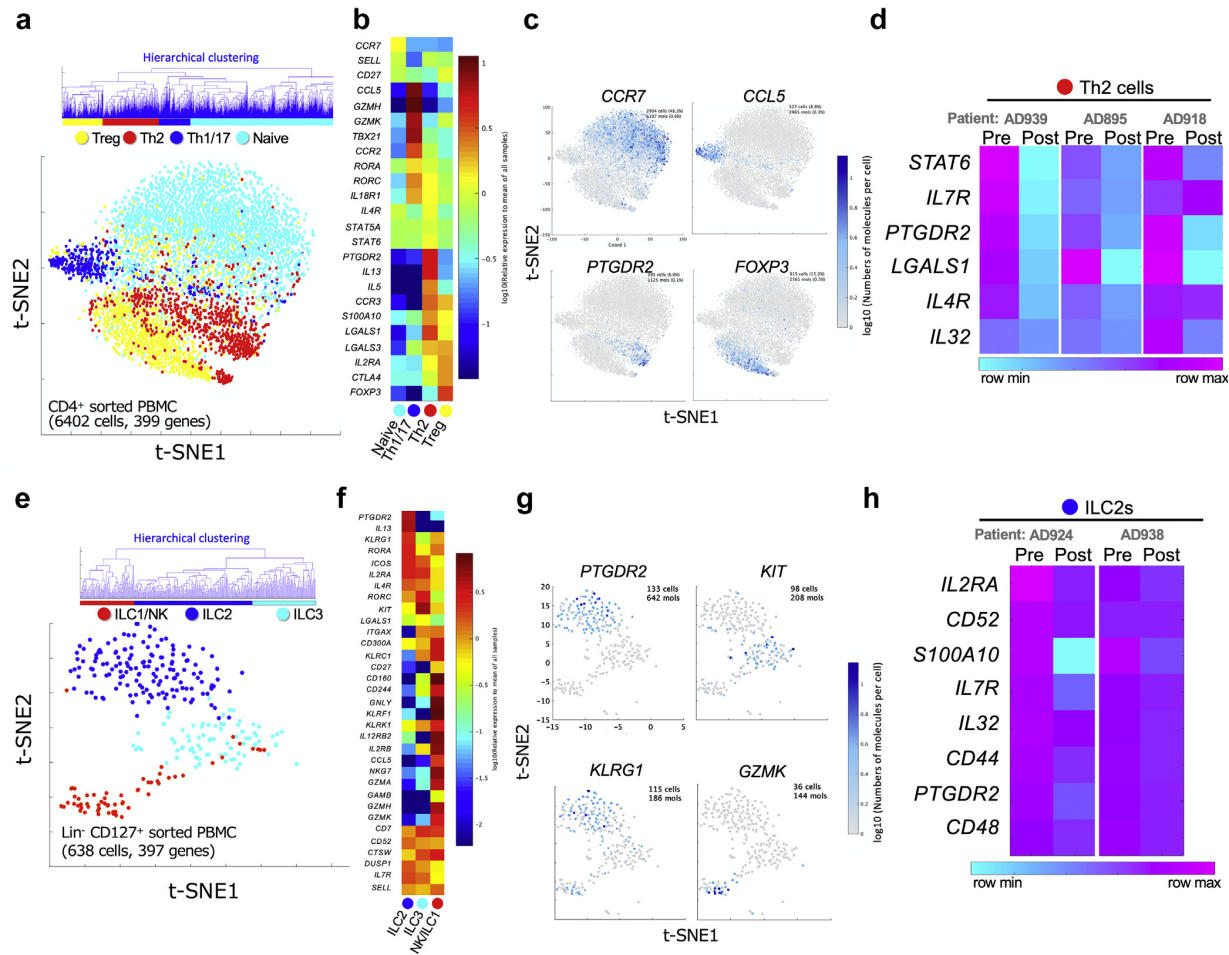
**Single-cell RNA-seq analysis of sorted CD4^+^ T cells and ILCs from human peripheral blood.** (**a**) Th2 cells are identified by single-cell RNA-seq. t-SNE plot of the single-cell RNA-seq data of 6,402 CD4^+^ T cells combined from six samples (before [Pre] and 16 weeks after [Post] dupilumab treatment from patient IDs 895, 918, and 939). Hierarchical clustering based on gene expression profiles was performed, and the T cells split into four clusters: naive T, Th1/17, Th2, and Treg. (**b**) Heatmaps of the representative genes from each cluster, naive T, Th1/17, Th2, and Treg. **(c)** Single-gene expression t-SNE plots of the single-cell RNA-Seq data. *CCR7*, *CCL5*, *PTGDR2* (CRTH2), and *FOXP3* are markers of naive T cells, Th1/17 cells, Th2 cells, and Treg cells, respectively. (**d**) Heatmaps of the representative genes (selected by differential expression analysis) gated on the Th2 cluster using the BD DataView software (n = 3, patient IDs 895, 918, and 939). **(e)** ILC2s are identified by single-cell RNA-seq. t-SNE plot of the single-cell RNA-Seq data of 638 ILCs combined from six samples (patient IDs 924 Pre/Post, 938 Pre/Post, 1049 Post, and 1062 Post). Hierarchical clustering based on gene expression profiles was performed, and the ILCs split into three clusters: NK/ILC1, ILC2, and ILC3. (**f**) Heatmaps of the representative genes from each cluster, NK/ILC1, ILC2, and ILC3. (**g**) Single-gene expression t-SNE plots of the single-cell RNA-seq data. *PTGDR2* (CRTH2), *KIT,* and *GZMK* are markers of naive ILC2s, ILC3s, and NK/ILC1, respectively. (**h**) Heatmaps of the representative genes (selected by differential expression analysis) gated on ILC2 cluster using the BD DataView software (n = 2, patient IDs 924 and 938). ID, identification; ILC, innate lymphoid cell; max, maximum; min, minimum; RNA-seq, RNA sequencing; Th, T helper type; Treg, regulatory T cell; t-SNE, t-distributed stochastic neighbor embedding.

Transcriptomic analyses of pretreatment and post-treatment bulk tissue skin biopsy specimens from patients with AD treated with dupilumab have been reported (Hamilton et al., 2014); however, these analyses did not distinguish the cell types. By contrast, single-cell RNA sequencing (scRNA-seq) is able to uncover cell-specific changes in gene expression. To resolve the molecular signatures of Th2 cells and ILC2s from patients with AD before and after dupilumab treatment, we sorted CD4^+^ T cells and ILCs from peripheral blood and analyzed their transcriptomes using the BD Rhapsody scRNA-seq analysis system (BD Biosciences, San Jose, CA) (Figure 3a–h) (Hasegawa et al., 2019). By using hierarchical clustering as described in Materials and Methods, T cells were split into four clusters, namely, naive T, Th1/17, Th2, and regulatory T cells (Figure 3a–c). Between 0 and 16 weeks after dupilumab administration, Th2 cluster gene signatures were remarkably changed (Figure 3d). ILCs cells were grouped into three clusters: NK/ILC1, ILC2, and ILC3 (Figure 3e–g). Between 0 and 16 weeks after dupilumab administration, ILC2 cluster gene signatures were downregulated (Figure 3h). These findings suggest that dupilumab administration affects both Th2 and ILC2 gene signatures.

## Discussion

There are a few reports of scRNA-seq analysis of AD skin (He et al., 2020; Rojahn et al., 2020), but none has identified cell clusters corresponding to Th2 cells and ILC2s because those studies analyzed all cell types, including keratinocytes, fibroblasts, and lymphocytes. Therefore, we solved this problem by initially sorting CD4^+^ T cells and ILCs and subsequently investigated isolated cells by using scRNA-seq.

The study has some limitations. We were not able to separate Th1 and Th17 populations by scRNA-seq analysis. Therefore, we used both flow cytometry and scRNA-seq analysis. The number of patients recruited is small, thus implying the need to validate our findings in a larger cohort of patients. In addition, future studies should investigate how ILC2 changes when AD is treated with anti–IL-13 antibody or Jak inhibitor.

Our results demonstrating dupilumab’s tendency to decrease Th2 cells and increase Th17 cells in the peripheral blood of patients with AD are consistent with those of a previous report (Trichot et al., 2021). We further showed by scRNA-seq analysis that dupilumab administration significantly altered Th2 cluster gene signatures. Increased frequency of circulating ILC2s in AD was previously reported (Mashiko et al., 2017); in vitro or animal experiments demonstrated that IL-4 is involved in the proliferation and activation of ILC2s (Imai, 2019). However, the actual mechanism has not been clarified in human AD. By comparing the number of ILCs in peripheral blood before and after administration of dupilumab in the same patient, we found that inhibition of IL-4 receptor signal reduces percentages and absolute numbers of ILC2s in peripheral blood of patients with AD. Furthermore, ILC2s were more depleted in high responders than in low responders to dupilumab (Figure 1e), suggesting that activation of ILC2s by IL-4 might be involved in human AD. ILC2s might be an indicator of the effect of dupilumab. By measuring ILC2/ILC3 ratio before administration of dupilumab, it may be possible to predict the effect of dupilumab (Figure 2c).

Thus, dupilumab might improve dermatitis by suppressing circulating Th2 cell and ILC2 populations and altering the Th2/Th17 or ILC2/ILC3 repertoire in patients with AD.

## Materials and Methods

### Patients

This study was conducted at Hyogo College of Medicine Hospital, Nishinomiya, Japan, to assess the effects of dupilumab in patients with moderate-to-severe AD who visited the hospital from April 2018 to March 2020. This study was approved by the Ethics Review Board of Hyogo College of Medicine (No. 0032 and 0212) and conformed to the Declaration of Helsinki. Blood was collected after obtaining written, informed patient consent. The diagnosis of AD was made according to the Hanifin and Rajka criteria.

### Definition of high and low responder to dupilumab

To analyze the factors dictating high or low clinical responsiveness to dupilumab, patients were subgrouped into two groups according to the rank order of percentage change of Eczema Area and Severity Index from baseline at week 16. Based on the classification used in previous reports (Nettis et al., 2020; Tavecchio et al., 2020), 75% of patients in this study were tentatively defined as high responders and the rest (25%) were tentatively defined as low responders (Figure 1a).

### Antibodies

Anti-human Lineage Cocktail-FITC (CD3, CD14, CD16, CD19, CD20, CD56; UCHT1, HCD14, 3G8, HIB19, 2H7, HCD56; #348801), anti-human CD123-FITC (6H6, #306014), anti-human FcεRIα-FITC (AER-37, #334608), anti-human CD45-PerCP/Cy5.5 (HI30, #304028), anti-human CD117-APC (104D2, #313206), anti-human CRTH2 (CD294)-PE/Cyanine7 (BM16, #350117), anti-human CD127 (IL-7Rα)-Brilliant Violet 421 (A019D5, #351309), anti-human CD183 (CXCR3)-PE (G025H7, #353705), anti-human CD4-PerCP/Cyanine5.5 (OKT4, #317427), anti-human CD196 (CCR6)-PE/Cyanine7 (G034E3, #353417), anti-human CD194 (CCR4)-APC (L291H4, #359407), and anti-human CD45-Brilliant Violet 510 (2D1, #368525) were purchased from BioLegend (San Diego, CA). Anti-CD16/32 antibody was from Miltenyi Biotec (Auburn, CA).

### Flow cytometry

We collected peripheral blood samples from the patients and isolated lymphocytes from human whole blood using a lymphocyte separation solution kit (#20839-04, Nacalai Tesque, Kyoto, Japan), according to the manufacturer’s instructions. Residual erythrocytes were lysed using ACK Lysing Buffer (Thermo Fisher Scientific, Waltham, MA). Cells were preincubated with anti-CD16/32 antibody for blocking and were subsequently stained with the appropriate antibody for 30 minutes at 4 °C. Stained cells were analyzed using a FACSAria III flow cytometer (BD Biosciences), and data were analyzed using FlowJo software (v10.5) (Tree Star, Ashland, OR). The classification of cells is as follows: ILC2s, lineage markers (Lin) (CD3, CD14, CD16, CD19, CD20, CD56, CD123, FcεRIα)^–^ CD45^+^ CD127^+^ CRTH2^+^ cells; ILC3s, Lin^–^ CD45^+^ CD127^+^ CD117^+^ CRTH2^–^ cells; total ILCs, Lin^−^ CD45^+^ CD127^+^ cells; Th2 cells, CD45^+^ CD4^+^ CCR6^−^ CXCR3^−^ CCR4^+^ cells; and Th17 cells, CD45^+^ CD4^+^ CCR6^+^ CCR4^+^ cells.

### scRNA-seq analysis

CD45^+^ CD4^+^ T cells or Lin^−^ CD45^+^ CD127^+^ cells were isolated from blood samples using a FACSAria III cell sorter (BD Biosciences). Targeted scRNA-seq analysis was performed using the BD Rhapsody Single-Cell Analysis System (BD Biosciences), according to the manufacturer’s instructions. For the library construction, we used the BD Human Single-Cell Multiplexing Kit (#633781, BD Biosciences) and the BD Rhapsody Immune Response Targeted Panel for Human (#633750, BD Biosciences), which consisted of primer sets for 399 genes. Sequencing was performed using an Illumina HiSeq X (Illumina, San Diego, CA), and the fastq files were converted using BD Rhapsody Analysis Pipeline (BD Biosciences) and analyzed using the BD DataView software v.1.2.2 (BD Biosciences).

### Hierarchical clustering

Hierarchical clustering is a method of cluster analysis that attempts to build a hierarchy of clusters. All analyses were performed in BD DataView software using MATLAB (R2014a, MathWorks, Natick, MA) (Joshi et al., 2017) built-in cluster function with the maxclust option, according to the manufacturer’s instructions. The specific formula is described elsewhere (Joshi et al., 2017; Zhang et al., 2018).

### Statistical analysis

Data were analyzed using GraphPad Prism version 8 (GraphPad Software, San Diego, CA). A two-tailed unpaired *t*-test was used for single comparisons. The Wilcoxon matched-pairs signed rank test was used for intragroup comparison. A *P*-value < 0.05 was considered statistically significant.

### Data availability statement

No datasets were generated during this study.

## ORCIDs

Yasutomo Imai: http://orcid.org/0000-0003-3169-5717

Minori Kusakabe: http://orcid.org/0000-0003-4422-4471

Makoto Nagai: http://orcid.org/0000-0003-3638-706X

Koubun Yasuda: http://orcid.org/0000-0002-3533-1702

Kiyofumi Yamanishi: http://orcid.org/0000-0003-0484-2320

## Author Contributions

Conceptualization: YI; Data Curation: MK, MN, YI; Formal Analysis: YI; Funding Acquisition: MN, YI; Investigation: KYas, MK, MN, YI; Methodology: KYas, YI; Project Administration: YI; Resources: MK, NM, YI, KYam; Supervision: KYam; Validation: KYas, YI; Visualization: MK, YI; Writing - Original Draft Preparation: YI, MK; Writing - Review and Editing: KYas, YI
